# International marine environmental governance: A review

**DOI:** 10.1007/s13280-016-0847-9

**Published:** 2016-11-15

**Authors:** Kjell Grip

**Affiliations:** 1 Department of Ecology, Environment and Plant Sciences, Stockholm University, 106 91 Stockholm, Sweden; 2 Mandelblomsgatan 11, 745 36 Enköping, Sweden

**Keywords:** Convention, International organization, Marine, Planning and management, Sustainable development

## Abstract

Impressive numbers of global and regional governmental and non-governmental organizations are working in the field of the marine environment and its resources. Many of these organizations operate within international legal frameworks ranging from comprehensive global conventions, such as the United Nations Convention on the Law of the Sea to regional agreements aiming at protection and development of regional seas. Characteristic for the management of these seas, both at the national and international level, is that sectoral approaches predominate. Over time, several initiatives have been taken to improve cooperation, coordination and integration to achieve greater coherence of policies and strategies between different organizations dealing with marine and maritime management, within and outside the United Nation system. However, the success has been limited. The weaknesses of international organizations depend fundamentally on problems at the national level. The international organizations are no stronger than their Contracting Parties allow them to be.

## Introduction

The seas and coasts are increasingly being used both to provide the basics of life and for commerce and recreation. The results include overexploited fisheries, pollution by pesticides, fertilizers and waste washed from land and overdeveloped coasts. In addition, the increasing effects of climate change are evident on ocean temperature, currents, food chains and extreme events.

Growing demand puts increasing pressures on the resources of the oceans and on governments to act, but short-term needs often limit their ability to adopt and implement effective long-term solutions

Measures against marine pollution or other threats to the marine environment will be more efficient if several countries work together, rather than each country is acting on its own (Abbott and Snidal 1998). There are many global and regional programmes that deal directly or indirectly with the protection and conservation of our seas, and the management of their resources. They cover a wide range of:

- *Research programmes* designed to improve our knowledge and understanding of the physical, chemical and biological processes that form the basis for maintenance and functioning of marine ecosystems, including social and economic developments and interactions with the atmosphere and the land;
- *Monitoring and assessment programmes* designed to monitor the status of the marine environment, including its resources and the changes taking place in the environment owing to natural and anthropogenic causes; and
- *Management programmes* designed to ensure the rational management and use of the seas and their resources.

These programmes assess global, regional and national environmental conditions and trends, develop international and national legal environmental instruments, and strengthen institutions working in the marine management field. Other elements in these programmes are to take appropriate legislative, administrative and other measures in accordance with their mandate and area of responsibility. The institutional framework of the programmes is impressive (See “Major marine institutional frameworks” section).

The aim of the present study is to provide a short review of the major global and regional environmental organizations and conventions relevant to the marine environment and the development of their work from the 1980s up to present, including examples from Europe and the Caribbean.

## MATERIALS AND METHODS

The present paper is based on information in various databases accessible at university libraries, and through Internet, including the Web of Science (ISI, Philadelphia). Personal experiences from international work at the Swedish Environmental Protection Agency and international organizations also underlie my studies, for example, the United Nations Environment Programme/Caribbean Regional Coordinating Unit (UNEP-CAR/RCU), the European Commission (EC), the Helsinki Commission (HELCOM), the OSPAR Commission (OSPAR) and the Nordic Council of Ministers.

The review: Provides information on the international legal^1^ frameworks related to the management of the marine environment, and describes the institutional^2^ frameworks of the major international (global and some regional) organizations in the United Nations system, Europe, and among inter-governmental and non-governmental organizations;

Examines ocean governance in practice, including marine and maritime management in the UN and EU systems, and the interactions between the described programmes and instruments for their coordination;

Analyses the organizational weaknesses and initiatives for promoting coordination and coherence (see Box 1), and highlights the challenges for marine and maritime management; and

Summarizes the final conclusions and suggestions for marine governance.

**Box 1:** The meaning of certain words

In the paper the words interaction, cooperation, coordination, integration and coherence are used in the following meaning:
Interaction: the situation or occurrence in which two or more objects or events act upon one another to produce a new or stronger effect.
Cooperation: the action when organizations are working or acting together for a common purpose or benefit.
Coordination: the process where organizations are organizing themselves so that they work together properly and well
Integration: the act where organizations are combining or adding parts of their work to make a unified whole. Integration is harder to achieve (See “The 1992 United Nations Conference on Environment and Development” section).
Coherence: a logical, orderly and consistent relation of different parts of for instance a strategy or policy addressed by several organizations

## Overarching legal and institutional frameworks of the sea

Most of the global and regional marine programmes are carried out in the scope of intergovernmental agreements, often in the form of international conventions. The United Nations Convention on Law of the Sea (UNCLOS) is a comprehensive global and legal instrument that can be regarded as an overarching framework for the many global and regional research, observation and management programmes (Churchill and Lowe 1999). In addition, the United Nations Conference on Environment and Development (UNCED) required new approaches to marine and coastal area management and development, at the national, sub-regional, regional and global levels.

### Legal frameworks

#### United Nations Convention on Law of the Sea: UNCLOS

In relation to targets, for instance, improved cooperation among international marine organizations on environmental standards, the decade of the oceans**—**the 1970s**—**is considered as a failure (DSH 1988; VanderZwaag 1996, own information; Joiner 2005). Instead of improving international cooperation among states, the 11th session of the third UN Conference of the Law of the Sea (UNCLOS III^3^) even worsened the global division of the oceans, which it was meant to resolve. It was not possible to negotiate away the competing national interests that lay behind all international cooperation among states (McRae 1984). Although UNCLOS III did not succeed in relation to its targets, the great merit of the conference was that it managed to straighten out and facilitate the daily intergovernmental administration regarding the use and protection of the oceans (DSH 1988).

**Fig. 1:**
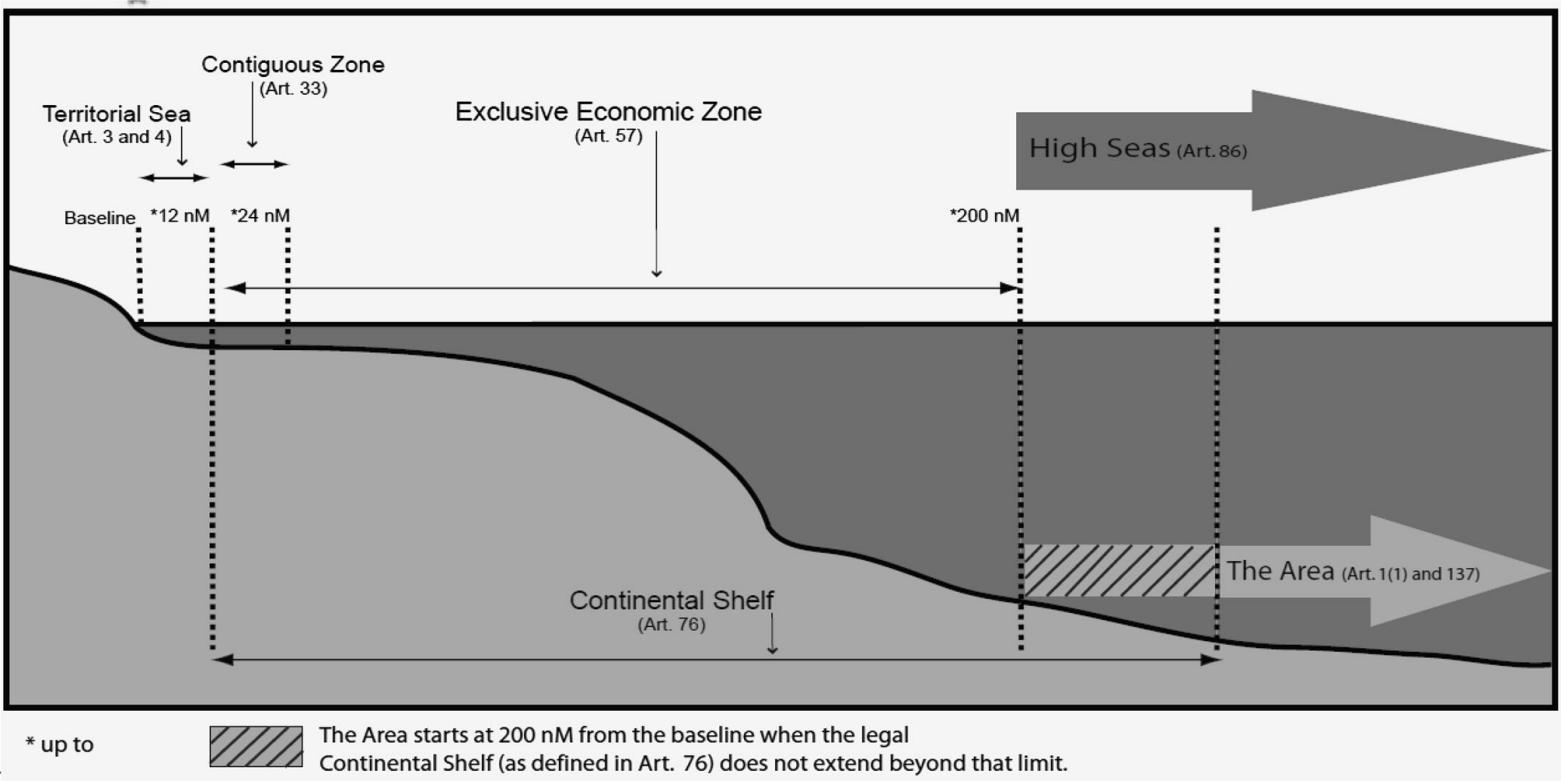
Graphical representation of UNCLOS and the boundaries at the Sea. Source: UNCLOS. The Sea is jurisdictionally divided into Inner Waters (inside the baseline), the Territorial Sea (12 nautical miles (nM) from the baseline), a Contiguous zone (a possible additional zone 24 nM from the baseline, claimed by some countries), the Exclusive Economic Zone, EEZ, (200 nM from the baseline) and the High Seas (beyond the EEZ). The Area includes the seabed and ocean floor and subsoil thereof, beyond the limits of national jurisdiction. It starts at the 200 nM or at the end of the Continental Shelf, where this extends beyond the 200 nM boundary (DSH 1983:1). Maritime boundaries delimiting various maritime zones in, for example semi-enclosed seas such as the Baltic Sea, are subject to special rules under UNCLOS

*The UN Convention on the Law of the Sea* (Fig. 1), adopted in 1982 and entered into force in 1994, provides the international and national legal marine framework needed in coastal countries for issues regarding their sovereignty, rights and responsibilities relevant to the management of the marine environment and its resources (Jacobsson 2009). Furthermore, UNCLOS includes a host of global agreements on specific issues, such as those related to management of fisheries resources, safety of maritime traffic, pollution control, protection and conservation of biodiversity, response to expected climate change, and to regional agreements aiming at protection and development of regional seas (Frank 2007).

According to UNCLOS, the state is the only “property owner” in the sea and it is the government that has the legal right and responsibility to issue licenses and permissions connected with the use and protection of the Sea under national sovereignty and jurisdiction (Jacobsson 2009). In most countries, this responsibility starts at the coastline.^4^ In coastal communities, especially those related to fishing, reference to traditional rights to living marine resources are common (Kearney et al. 2012). Protests by local fishing communities against infringements of these rights are frequent and fervent, for instance, against restrictions on fishing in a marine protected area or wind park (Redpath et al. 2015). However, compensation for restriction of the “traditional rights” to fishing differs from that of a landowner, whose forest cannot be felled.

The creation of the High Seas as a common pool resource did not take into account the effect this “common area” would have on migratory marine species. There are still gaps in the regulation, for instance, of fisheries in the High Seas (FAO 2007). Also, there is no real consideration of the fact that valuable marine resources regularly transit between the High Seas and zones of national sovereignty. UNCLOS lacks enforcement measures with sufficient incentives for state actors to collectively act for the conservation of marine species in general and migratory marine species in particular (Baker et al. 2001; de Fontaubert 2001; Mc Guire 2003).

With regard to nature conservation in the High Seas, a network of six High Seas Marine Protected Areas was established in 2010 by OSPAR in coordination with the North East Atlantic Fisheries Commission (O´Leary et al. 2012). These are zones or areas in the High Seas where resource extraction is prohibited for conservation purposes. However, in order to be efficient, marine nature conservation in the High Seas needs to be equipped with adequate enforcement mechanisms under UNCLOS in a similar way as, for example, the Straddling Fish Stock Agreement (de Fontaubert 2001; Mc Guire 2003). Currently, there is strong resistance against such measures from some countries that argue for the freedom of the Seas, and it will take time before proper protection and sustainable use of living resources in the High Seas can be achieved. The use of High Seas marine protected areas has yet to be incorporated formally into international law (Corrigan and Kershaw 2008; Houghton 2014).

#### The 1992 United Nations Conference on Environment and Development

*The 1992 UN Conference on the Environment and Development (UNCED)* in Rio de Janeiro, added a new theoretical overlay to the 1982 UNCLOS (VanderZwaag 1996, own information). Agenda 21, the Programme of Action for Sustainable Development, involves issues on interaction between governments, intergovernmental and nongovernmental organizations. The conference sets out a number of principles calling for new approaches to the governance and management of the use, protection and conservation of natural resources. According to VanderZwaag (1996), perhaps the most powerful outcome of the Rio Conference was the new emphasis on principled decision-making. Through Agenda 21, UNCED articulated several important principles of sustainable development, such as integration, precaution, pollution prevention, intergenerational equity, polluter pays principle, public participation, community-based management, indigenous rights and women and development. Three of these principles are of special importance for the governance and management of the marine environment namely—precaution, integration and community-based management (Kubiszewski and Cleveland 2012).

The 1992 UNCED conference left numerous issues unresolved. Among these were the meaning and implementation through law of the principles of sustainable development, and the need to strengthen the commitments pursuant to processes established by the Rio Conventions. Both UNCLOS (III) and UNCED articulated several “soft law” principles^5^ that should guide international, as well as national law and policy reforms with regard to coastal and marine management (VanderZwaag 1996, own information; Jacobsson 2009). However, while UNCED recognized the problem of coordination and integration, it failed to improve the efficiency of international cooperation in maritime activities, for instance, between fisheries and nature conservatison (Grip 2003; Redpath et al. 2015).

The 2012 review of the Rio Principles (Dodds et al. 2012) shows that while many of them have been transposed further into international laws or national instruments, they have not necessarily filtered down into meaningful action in practice. Without full compliance and enforcement mechanisms, there is little to ensure that States comply with the objective and aspiration of the principles. While the precautionary principle has been rather widely accepted, the legal implementation of various forms of community-based management or local governance over marine resources has, according to the review, so far been slow. Likely, one reason is that in most countries, the power of management of the Sea belongs to the state and transfer of power often meets resistance (Kearney et al. 2012).

According to VanderZwaag (1996), the term integration is perhaps one of the most loosely used words in the ocean management field, but also a key principle for sustainable development. The term may refer to the need to:

- integrate environmental and socio-economic considerations in all decision-making sectors;
- overcome fragmentation in authorities responsibilities and permitting/licensing processes;
- adjust management arrangements to reflect ecosystem realities;
- overcome conflicts of uses in a particular area; and
- interdisciplinary integration of different types of knowledge.

According to Dodds et al. (2012) review, the implementation of the integration principle has been limited or slow in most countries. An obvious reason is competing interests between different activities such as fishery, nature conservation and shipping, and that these sectors already have their own legal frameworks implemented by different independent authorities and backed up by different economic interests. However, integrative steps have been taken. In 2014, for example, the European Union Directorate-General (DG) Environment and DG for Maritime Affaires and Fisheries (MARE) was merged to one portfolio—DG for Environment, Maritime Affaires and Fisheries (EC Press Release IP/14/984) (See “Marine and maritime management in the EU” section).

#### Agenda 21 and the marine environment

The chapter 17 of Agenda 21 (UNCED 1992) was devoted for protection of the oceans and seas. It reiterated the key principles of sustainable development and introduced seven programme areas for priority action:

- Integrated management and sustainable development of coastal and marine areas;
- Marine environmental protection;
- Sustainable use of marine living resources of the High Seas;
- Sustainable use and conservation of marine living resources under national jurisdiction;
- Addressing critical uncertainties for the management of the marine environment and climate change;
- Strengthening international, including regional, cooperation and coordination; and
- Sustainable development of Small Islands Developing States (SIDS).

In addition to Agenda 21, the Conventions on Climate Change and Biological Diversity (CBD) were adopted in Rio. The CBD is a comprehensive, binding agreement covering the use and conservation of biodiversity. However, the 1992 document lacked specific articles on marine and coastal biodiversity. Instead, the Jakarta Mandate is the global consensus on the importance of marine and coastal biological diversity. This mandate is part of the Ministerial Statement at the second meeting of the Conference of the Parties (COP) of the CBD (CBD/COP 2) in Jakarta 1995. Its work programme was adopted at the CBD/COP 4 meeting 1998 (CBD 2000).

Also at CBD/COP 4, the 12 Malawi principles for the ecosystem approach^6^ on the management of land, water and living resources were presented (UNEP/CBD/COP/4/Inf.9). The term is often used synonymously with ecosystem-based management^7^ but there is a difference. Ecosystem-based management is a governance instrument with an integrated approach that considers the structure and function of the entire ecosystem, including humans, with the goal of maintaining healthy, resilient and productive ecosystems that can provide goods and services.

As a result of the Rio conference, manuals and guidelines for Integrated Coastal Area Management (ICAM) were produced by a number of organizations, inside and outside of the UN system (UNEP/CEP 1996), and by individual countries (NRCA 1997). ICAM or Integrated Coastal Zone Management and Marine or Maritime Spatial Planning (MSP) have become important management instruments for integration and inter-sectoral coordination of the sustainable use of coastal marine waters and lands, as well as open marine waters. Today, these instruments and the principles of ecosystem-based management have been further developed, for example, by the EU, HELCOM, OSPAR and UNEP Regional Seas Programmes (RSPs) (Pickaver 2002; HELCOM 2003a; Douver 2008; OSPAR 2010a).

At UNCED 2012, also in Rio de Janeiro (United Nations 2012), many countries requested that a “Blue economy process” should be more properly addressed in the context of sustainable development within the UN Green economy concept. This request is reflected in the prominence given to oceans and seas in the UN 5-year Action Agenda 2012–2016 (UNEP 2012) and the EU Blue Growth strategy (EU/EC 2014). The Blue Economy approach also recognizes and emphasizes the need for efficient planning and management of the use, protection and conservation of coastal and marine resources, as well as the further development of international law and ocean governance mechanisms, such as marine spatial planning and the ecosystem approach. In the last decade, marine spatial planning has gained considerable importance in establishing ecosystem-based management in the marine environment (Douvere 2008). Today, the ecosystem approach is commonly featured in marine policy documents, but managers still struggle with its interpretation and practical implementation (Farmer et al. 2012; Elmgren et al. 2015). In this context, it is worth reminding that ocean management is not limited to the national level (Vallega 1993).

### Major marine institutional frameworks

#### Global marine-related organizations within the United Nations system

Two United Nations bodies, the *Intergovernmental Oceanographic Commission* (*IOC*) and the *International Maritime Organization* (*IMO*) are exclusively concerned with ocean affaires. IOC promotes marine scientific investigations, and IMO is dealing with shipping and pollution from maritime activities. Several other UN bodies have ocean-related issues among their core activities. One is the *United Nations Environment Programme* (*UNEP*) that one year after its creation (1973) selected the protection and development of oceans and coastal areas as one of its six major programme areas, and launched the *Regional Seas Programme* (*RSP*) (Keskes 1997, own information).

Other marine-related UN bodies are the *Food and Agriculture Organization* (*FAO*), with its subsidiary body *Committee on Fisheries* (*COFI*), the *World Meteorological Organisation* (*WMO*) dealing with global climate, the *United Nations Educational, Scientific and Cultural Organization* (*UNESCO*) dealing with marine sciences, *United Nations Conference on Trade and Development* (*UNCTAD*) dealing with technology transfer, and the *International Seabed Authority* (*ISBA*) with responsibility for mineral resources of the seabed.

IMO, FAO, WMO and UNESCO are specialized autonomous agencies with their own budgets and status, while UNEP is subordinate to the UN General Assembly, and the IOC is subordinate to UNESCO with budgets controlled by their mother organizations. Interestingly, in the 1980s, merging the marine activities of UNEP, IOC and COFI of FAO in a new organization, the *International Ocean Agency* (*IOA*) (DSH 1988) was discussed but not realized.

#### Intergovernmental and non-governmental organizations outside the UN system

There are also many intergovernmental and non-governmental organizations (NGOs) outside the United Nations system that play important roles in promoting global and regional marine-related research and management.

Among the most prominent intergovernmental organizations are the *International Council of Scientific Unions* (*ICSU*) promoting international cooperation and coordination in the advancement of science, the *International Council for the Exploration of the Sea* (*ICES*) concerned with marine and fisheries sciences, and scientific advice on marine and fisheries management to regulatory commissions, the *European Commission* (*EC*) is the executive body of the European Union (EU), the *International Union for Conservation of Nature* (*IUCN*) provides a forum for governments and NGOs to discuss global and regional conservation issues and the *International Bank for Reconstruction and Development* (*World Bank*) promoting the flow of capital internationally by lending funds for development projects.

Among the many different types of environmental NGOs involved in both global and regional marine issues are the *World Wildlife Fund* (*WWF*), *Greenpeace*, *Oceana*, *Birdlife International* and *Seas at Risk* (an umbrella organisation of environmental NGOs from across Europe) can be mentioned (See “The role of non-governmental organizations” section). Oceana is the largest NGO focused solely on ocean conservation, protecting marine ecosystems and endangered species. Example of another kind of NGO is the *Regional Advisory Councils* (*RACs*) connected to the work of the EU Common Fisheries Policy (CFP). The RACs involve different stakeholders, such as fishermen, vessel owners, processors, traders, fish farmers, women’s fisheries groups, environmental and consumer organizations and others. Their role is to submit opinions to the European Commission and Member States on different aspects of fisheries management. Other examples in Europe are *Europeche* (representing fishermen) and *Euro Chlor* (representing the chloralkali industry). Several indigenous NGOs act at regional and local levels. In Australia, for example, indigenous peoples’ rights and interests in marine protected areas have recently been recognized (Ross et al. 2009). In the Arctic Council (see below), seven indigenous communities are permanent participants of the Council.

#### Regional marine-related organizations in Europe

International commitments regarding regional European seas are mainly connected to the regional marine conventions: the *Helsinki Convention on the Protection of the Marine Environment of the Baltic Sea Area* (*HELCOM* 8), the *Convention for the Protection of the Marine Environment of the North-East Atlantic* (*OSPAR* 9), the *Convention for the Protection of the Mediterranean Sea Against Pollution* (*Barcelona Convention*) *and the Convention on the Protection of the Black Sea Against Pollution* (*Bucharest Convention*). These organizations are the regional focal points for environmental protection and nature conservation in their respective sea areas.

Within the legal framework of IMO, the regional marine commissions and their Contracting Parties, coordinate (e.g. the Helsinki Commission) or cooperate through joint activities (e.g. UNEP/RSPs and the OSPAR Commission) to protect the regional seas against pollution from ships and other maritime activities. The *International Council for Exploration of the Sea* gives, as mentioned above, scientific advice on marine environment and fisheries management to regulatory commissions and the EU. The *Nordic Council of Ministers* (*NCM*) covers a much wider area than just the marine environment but has a marine environmental working group—the Nordic Marine Group. It contributes to the implementation of relevant marine NCM activities, such as the Environmental Action Programme 2013–2018 (NCM 2012) and the Arctic Cooperation Programme. The *Arctic Council* promotes coordination and interactions among the Arctic states and their indigenous communities.

None of these organizations have a mandate to work with marine environmental issues in a comprehensive way. ICES are responsible for the coordination and promotion of marine scientific research, and on request, provide scientifically based advice within the area of the environment and fishery, for example, to HELCOM, OSPAR and the EC. The regional marine environmental commissions deal with the environmental effects of fishing, but the fishery is managed by the regional fishery commissions under FAO and the European Union (EU). Within the EU, the Common Fisheries Policy and the Maritime Transport Policy are the coordinating mechanisms for fisheries and shipping, respectively (Salomon 2009). The Marine Group of the Nordic Council of Ministers sometimes coordinates common Nordic issues within the work of, for example, HELCOM, OSPAR and the EU (NCM 2012).

### The regional marine commissions and the EU

The European Union is a contracting party to HELCOM, OSPAR and the UNEP/MAP Secretariat for the Barcelona convention, which at the regional level coordinate and facilitate the implementation of EU directive requirements, especially the Marine Strategy Framework Directive (MSFD). The EU and UNEP are observers to the Bucharest convention.

At the end of the 1990s and the early 2000s, several Contracting Parties to the regional marine conventions in northern Europe gave a lower priority to the work in HELCOM and OSPAR (Kern and Loffelsend 2004; Valman et al. 2014). After the Soviet Union collapsed in 1991, and the expected enlargement of the EU, more focus was put on its marine work and the development of a European Marine Strategy. For example, the Swedish Environmental Protection Agency (SEPA) considered that Swedens international marine work should give priority to the EU. The work with HELCOM and OSPAR was to be reduced, and SEPA would no longer assume a lead country role in the commissions (Naturvårdsverket 2004).

In 1999, the work of HELCOM was reviewed and restructured without changing the convention (HELCOM 1998a, own information). HELCOM was not longer a forum for East–West bridge-building, as during the era of the Soviet Union. HELCOM’s role in the Baltic Sea framework had become uncertain, partly as a consequence of the further enlargement of the EU. Today, the European Commission coordinates its work with the marine regional commissions as the most appropriate way to protect the regional marine environments and their resources.

Following the Bremen Declaration from the joint HELCOM and OSPAR Ministerial Meeting in Bremen 2003, cooperation and coordination with the EU, other international bodies and regional seas conventions became increasingly important (HELCOM 2003b). This strengthened the role of the regional marine commissions, and today work is continuous on coordination and harmonization, for example, of HELCOM recommendations and OSPAR decisions with EU’s marine-related directives, especially the MSFD and the Birds and Habitats Directives. The HELCOM and OSPAR strategic goals are largely compatible with the MSFD criteria for achieving Good Environmental Status by 2020 (See “Marine and maritime management in the EU” section), which according to the Baltic Sea Action Plan should be achieved by 2021(HELCOM 2007). Fisheries management remains under the EU Common Fisheries Policy, but the environmental effects of the fishery are addressed by the regional marine commissions.

### Expansion of marine-related organizations and their activities

Programmes and activities in the marine sector expanded notably after the UNCED Conference in Rio de Janeiro in 1992 with increasing demands on integration across boundaries and sectors. No organization wanted to be left behind in demonstrating its relevance to Agenda 21. The many manuals and guidelines for ICAM produced by a number of organizations inside and outside the UN system are an example (Keskes 1997, own information).

#### From pollution prevention to a broader approach

Initially, the regional marine environmental conventions and their commissions mainly dealt with marine pollution. Much as a response to the 1992 Rio conference, the new 1992 Helsinki and OSPAR conventions also began to address issues of biodiversity, marine protected areas and sustainable development (HELCOM 1996, 1998b). In 1995, the Barcelona convention adopted a new protocol on protected areas and biodiversity, and in 1998, OSPAR adopted a new Annex on Biodiversity and Ecosystems.

HELCOM and OSPAR have established a joint and ecologically coherent network of Marine Protected Areas (MPAs), in line with the EU Natura 2000 network and in accordance with the Birds and Habitats Directives (HELCOM 2003b, 2010; OSPAR 2003, 2010b). Still, many MPAs in the network are only designated, lack management plans and are not fully protected. Similar regional MPA networks have been established in the other UNEP/RSPs (UNEP-WCMC 2008) and, for instance, in Australia (Fernandes et al. 2005) and the USA (Gleason et al. 2010).

#### New initiatives

Also, as a response to the 1992 Rio Conference, new organizations turned up with an agenda that the existing organizations already had a mandate to deal with. In the Baltic Sea region, organizations such as *Vision and Strategies around the Baltic Sea* (*VASAB*) and *Baltic 21* (a regional process for cooperation on sustainable development) are examples of “overarching” initiatives that were added to other already existing programmes in the Baltic Sea region, for example, HELCOM. In 2010, Baltic 21 was incorporated in the *Council of the Baltic Sea States.* Today, VASAB is an intergovernmental multilateral cooperation of 11 countries in the Baltic Sea Region that focusses on spatial planning and development, including marine spatial planning, and cooperates with HELCOM on implementing the Baltic Sea Action Plan (HELCOM 2007; Valman 2014).

At the global level, the many regional seas programmes, UNEP’s Global Programme of Action (GPA) for the Protection of the Marine Environment from Land-based sources, and the “new” marine research programmes of UNESCO and IOC that started, are other examples of activities that often overlapped with existing programmes and were underfunded (Keskes 1997, own information). For instance, the GPA in the beginning suffered from both financial and manpower resources. Often its programme was not coordinated and it competed with already ongoing programmes in the RSPs and caused confusion. However, that does not mean that GPA projects have later on not been properly coordinated and successful.

Sometimes the work of existing organizations has been criticized, and new programmes proposed at the political level. It seems it was easier to create a new organization instead of giving an existing one the support and funds needed to do the work properly. An example of such a new programme in the Baltic Sea region was the Joint Comprehensive Programme (JCP), managed by a special body within HELCOM, the Programme Implementation Task Force (HELCOM PITF). This body was set up to provide funds for solving the environmental problems of the Baltic Sea, and restore it to good ecological status, a task that existing HELCOM bodies were, in fact, already working on. PITF had as members the Contracting Parties of HELCOM plus representatives of international financial institutions (the European Bank for Reconstruction and Development, the European Investment Bank, the Nordic Environment Finance Cooperation, the Nordic Investment Bank and the World Bank). PITF was active during the 1990s, and closed down in 2003.

HELCOM PITF addressed significant pollution sites (hot spots) and made management plans for sensitive coastal lagoons and wetlands around the Baltic Sea. Apart from investments activities, the HELCOM PITF essentially addressed tasks that other HELCOM committees were mandated to handle—legal and regulatory measures, institutional strengthening, applied research and public awareness. HELCOM PITF dealt with too many things, lacked proper coordination with the work of the other relevant bodies of HELCOM and sometimes caused confusion and duplication of work, e.g. on monitoring and assessments (Grip 1999, own information).

A similar example is the Conference on North Sea Senior Officials (CONSSO). This currently “sleeping” organization was active from the 1980s up to 2006. In essence, CONSSO addressed the same North Sea issues as OSPAR but limited to the North Sea. The latest CONSSO conference, chaired by Sweden, focussed on the environmental effects of shipping and fishing (CONSSO 2006), even though CONSSO lacked the mandate to manage shipping and fishery. The success of CONSSO was that the North Sea countries could address their common North Sea problems without having the whole OSPAR North East Atlantic region involved. Today OSPAR is managing the issues of CONSSO.

## Ocean governance in practice

International laws and organizations do not guarantee good governance,^10^ but can provide a basis for responsible and effective management^11^ by individual countries. In this context, it should be noted that competing national interests is usually behind all bilateral and multilateral cooperation and coordination through international organizations (Katsenavakis et al. 2011). Also, inter-organizational integration of policies and programmes is more difficult to achieve than cooperation and coordination between organizations (See “The 1992 United Nations Conference on Environment and Development” section). Furthermore, most intergovernmental agreements, even legally binding ones, are full of imprecisions, lack effective enforcement procedures and, in fact, are less binding than they purport to be.

### Marine and maritime management in the UN system

Marine^12^ environmental management within the UN system is of byzantine complexity. The research and observation programmes aim to fill the existing knowledge and data gaps, and improving the rather low predictive capability of the marine sciences for managing the oceans and seas, and their resources (Richardson and Poloczanska 2008). For this purpose, the end users of the programmes need to be adequately involved in their design, development and implementation. Management programmes for regulation of maritime^13^ traffic and its environmental impacts have been rather successful (McConnell 2002). However, this cannot be said for the multi-facetted programmes dealing with marine pollution control, integrated management of coastal areas and, in particular, the management of fisheries (Beddington et al. 2007).

In spite of agreements and rules, there are many coordination problems and conflicts between the organizations involved in ocean and sea use management. Each agency basically pursues its own programme and defends its mandate. In the conservation field, such tensions and sometimes conflicts can be found between, for instance, UNEP/RSPs and FAO/Regional Fishery Organizations on environmental impact of fisheries and marine protected areas, and between UNEP/RSPs and IMO on environmental effects of shipping (Redpath et al. 2015). The inter-organizational cooperation and coordination depends, to a large extent, on the personal relationship and interaction between the staff “controlling” the programmes on behalf of their organizations (Own information). The efficiency of the UN system has been questioned with cause, but despite its shortcomings, the UN system will continue to play a central role in the environmental protection and the resource use management of the oceans and seas (United Nation 2012).

### Marine and maritime management in the EU

At the European level, there is no single policy or set of policies to manage the marine environment. Instead, there is a complex web of interacting and overlapping policies that leave significant problems unaddressed. The EU Blue paper on a *European Maritime Policy* (EU/EC 2007) is a strategy for a more optimal sustainable development of all maritime activities. By better integration of the different marine-related activities, a more coherent maritime policy should be created among marine-oriented policy areas, such as fishery, transport, environment, energy, industry, defense and science policies.

By the development of the maritime policy, the European Commission has established a maritime policy function, which aims to coordinate socio-economic issues related to the sea with marine environmental issues (Farmer et al. 2012). A corresponding policy is found in the UN Blue economy concept (UNEP 2012) (See “The 1992 United Nations Conference on Environment and Development” section). The Integrated Maritime Policy is a holistic approach to all EU maritime activities and policies. Its main instrument for coordinating the maritime policy spatially with various activities at sea is the *Framework Directive for Maritime Spatial Planning* (*Directive 2014/89/EU*).

With time, MSP has emerged as one important coordinating instrument for marine and maritime planning and management, and to achieve ecosystem-based sea use management (Douvere 2008; Farmer et al. 2012). Today, the MSP Directive is a cornerstone of the Commission’s *Blue Growth Strategy* (EU/EC 2014) and the Integrated Maritime Policy. This strategy contributes to a more efficient implementation of EU: s environmental legislation in marine and coastal waters. Several member countries already have or are now introducing MSP instruments for marine waters under national jurisdiction. Through the European Territorial Cooperation with a number of Interregional and other projects, the EU has financially supported the development of MSP, for instance:

- the Baltic Scope project on transboundary Baltic maritime spatial plans leading to greater alignment of national plans;
- the Balance project on Baltic Sea management for nature conservation and sustainable development of the ecosystem through spatial planning;
- the BaltSeaPlan for introducing maritime spatial planning in the Baltic Sea (HELCOM and VASAB); and
- the TPEA project on Transboundary Planning in the European coastal Atlantic states.

The *Marine Strategy Framework Directive* (*Directive 2008/56/EC*) is the environmental pillar of the *EU Maritime Policy*. The Marine Directive aims to deliver a coherent policy to meet, for the first time, the goal of good governance through legally binding targets and achieve *Good environmental status by 2020* (See “The regional marine commissions and the EU” section). The Marine Directive fills a gap in EUs environmental policy, which was earlier focused on land and freshwater issues. The directive is not only about pollution, but also covers the protection of species and habitats, and sustainable use of marine areas and their resources (see also Box 2).

**Box 2:** Other EU directives and regulations

Other EU directives and regulations which have or will have a significant influence on the management of the European seas are:
the *Water Directive* (*Directive 2000/60/EC*). The need to coordinate the implementation and monitoring of the *Marine Directive* with the *Water Directive* has specifically been emphasized;
the *Urban Waste Water Treatment Directive* (*Directive 91/271/EEC*);
the *Nitrate Directive* (*1991*);
the *Common Agricultural Policy, CAP* (*1962*);
the *Common Fisheries Policy, CFP* (*Treaty of Rome 1957*);
*REACH* (*Registration, Evaluation, Authorization of Chemicals*) (*Regulation 1907/2006 EG*).

Together the MSFD and the Maritime Policy with the MSP Directive should provide to a more coherent European maritime policy (Wanfei and Jones 2013). However, it should be noticed that both the environmental and maritime policies aim at governing the marine environment. They differ in focus between economic and ecological aims, and have different stakeholders and different ways of setting rules (Van Hoof and Van Tatenhove 2009).

In the early development of the MSFD under DG Environment, the suddenly presented proposal for a maritime strategy, by the former DG MARE, created some confusion and concern. There were clear tensions between the two directorates regarding the ambitions on blue growth and productive seas on one hand, and healthy and clean seas on the other (EEA 2015). These tensions remain, but in 2014, its new president restructured the European Commission by merging DG Environment and DG MARE (See “The 1992 United Nations Conference on Environment and Development” section). It remains to be seen whether this will create a more coherent maritime policy in practice.

### The role of non-governmental organizations

A NGO, also often referred to as “civil society organization” (or CSO) is a not-for-profit group, principally independent from government, which is organized on a local, national or international level to address issues in support of the public good. Environmental NGOs and pressure groups of different kind exist in many countries (See “Intergovernmental and non-governmental organizations outside the UN system” section), and their involvement in international and national marine environmental issues is important (Richards and Heard 2005). The role of NGOs was enhanced by the 1992 Rio Conference. Today, many environmental and other NGOs have observer status under the major international agreements, including the EU (Princen and Finger 1994). Occasionally, they exert decisive influence on marine-related policies and practices of individual countries. In contrast to individual states, which often pursue what they see as “national interests”, NGOs of different kind often bring a much needed broader “global and regional perspective” to the issue under consideration.

## Discussion

### Deficiencies in the management of the seas

At a national level, most countries still lack a coherent integrated policy for marine and maritime affairs. In most governments, there is a strong sector-oriented division among the different ministries, where different inter-ministerial coordination problems also are reflected in the cooperation between subordinate sector-authorities (Browman and Stergiou 2004). Weak cross-sector integration and conflicts at national level hamper a countries’ ability to act coherently at the international level.

The most obvious shortcoming of international organizations and national authorities is the fragmentation and lack of coordination between different programmes and institutions. The management deficiencies identified already in the Joint Group of Experts on the Scientific Aspects of Marine Environment Protection in their report *A Sea of Troubles* (GESAMP 2001) are to a large extent still present. This applies particularly to the feeble governance, including the failure to address their interlinked environmental problems in an integrated way and the weak influence on impacts from land-based activities.

The existing institutions and structures charged with the coordination of national marine environmental policies are in many cases too fragmented, and deal with the problems as sectoral issues, rather than as part of a coherent national marine policy (Brown et al. 2002). This applies to marine sectors such as fishery, logistics, environment and energy. As an example, there is a long history in almost all areas of the world of conflict and lack of cooperation between environmental and fisheries management agencies on what should be protected in a MPA—the ecological value of a strictly protected area or the economic value of a protected area regulated or open for fishery or a wind power establishment? (Kearney et al. 2012; Johannesen and Lassen 2014; Redpath et al. 2015). The relationship between agencies responsible for the management of the environment and shipping interests is similar. These institutions have their own sector legislation, and usually lack adequate authority to regulate and enforce environmental policies, or to influence national economic strategies, on which ultimately the protection and the development of the marine and coastal environment depends (Coleman et al. 2004).

Although politicians’ resolution to act is important, weakness in national institutions, policies and practices—all of them largely embedded in domestic and international economic and financial circumstances—seems to be the main reason for the generally inadequate national marine and maritime management programmes. These are many and governed by different authorities, which usually are highly interdependent. Even in the few countries where such programmes do exist, they are fragmented, managed in an uncoordinated way and implemented in a permissive manner (Frank 2007). The implementation of internationally adopted environmental action programmes and agreements requires action at the national level (Abbott and Snidal 1998).

### Initiatives promoting coordination and coherence

The major marine and maritime management programmes of the UN system are handled by IMO, FAO (fisheries) and UNEP/RSPs. Together with the research and observation programmes of IOC and WMO they represent the main part of the marine-related programmes in the UN system. Over time, several initiatives have been taken to improve cooperation, coordination and integration (see Box 1) in order to achieve increased coherence between different UN bodies, and other organizations dealing with marine and maritime issues, for example:

- Already in 1993, the UN Agencies dealing with oceans and coastal issues formed the *Sub*-*committee on Oceans and Coastal Areas of the Administrative Committee on Coordination* (*ACC SOCA*) in order to support and follow up on Chapter 17 of Agenda 21;
- In 2003, the United Nations High-Level Committee on Programmes approved the creation of an Oceans and Coastal Areas Network, named *UN Oceans* (United Nations 2003);
- The *UN Oceans Compact* is an initiative aimed at improving coordination related to oceans in the UN system and supporting the UN in delivering on its ocean-related mandates, consistent with the Rio+20 outcome, in a more coherent and effective manner. In January 2012, the United Nations Secretary-General launched the UN Ocean Compact “Five-Year Action Agenda” for a new UN Ocean Compact action plan (United Nations 2012);
- Another important initiative is the *Regular Process.* At the World Summit on Sustainable Development, held in Johannesburg 2002, states agreed to “establish by 2004 a regular process under the UN for global reporting and assessment of the state of the marine environment, including socio-economic aspects, both current and foreseeable, building on existing regional assessments” (United Nations 2004); and
- Yet another example of fruitful cooperation and coordination in marine environmental management is between the EU and the regional seas conventions and other international bodies, the Bremen declaration (HELCOM 2003b). The EU Directives are not binding for the regional commissions—only for EU member States. However, when EU Directives (e.g. MSFD) refer to work carried out in the regional marine commissions, this gives an extra legal impetus to the work of regional commissions, such as OSPAR, HELCOM and the UNEP/MAP Secretariat for the Barcelona convention. The EU has strengthened the role of the regional marine commissions for those EU Members, who are also Contracting Parties to the commissions.

### Present challenges for marine and maritime management

Marine and maritime management is by tradition characterized by sectoral management (Crowder and Norse 2008; Douvere 2008) and marine managers have always had limited impact on land management in coastal areas and river basins. Furthermore, the management of our seas is not only a national issue but need, to be effective, cooperation and coordination with other countries, usually through international organizations (Agenda 21/Chapter 17).

In deciding on the appropriate balance between environmental and development goals, marine and maritime managers need knowledge from many disciplines, such as sociology, engineering, political science, law, economics and ecology. It is essential in order to understand management constraints and provide a nuanced description of the factors that contribute to the outcomes in these systems, for instance, regarding the sustainable use of marine resources (Ostrom and Cox 2010; Epstein et al. 2013; Villasante et al. 2013).

However, the sectoral management and decision-making have not been sufficiently coordinated and integrated across various political and sectoral interests. Little consideration have been taken of how efforts to attain a goal in one sector would affect, or be affected by, efforts in another sector, or whether the total demand for key resources could be met by existing supplies without degrading the resource base and underlying ecosystems.

There is today an emerging paradigm shift in ocean management, towards consideration of the impacts of all ocean sectors on the marine environment, both separately and in aggregate. This comes from an increasing awareness of the cumulative effects of human activities on the ecosystems, and increasing resource and user conflicts over sectoral and political boundaries. Measures for improved marine and maritime management require the development, use and implementation of national legal frameworks, including instruments such as MSP and ecosystem-based management, as well as cooperation through and support by international organizations. This, in turn, requires a responsible coordinating authority function that can take care of, investigate and shed light on problems that are related to several different marine sectors or areas of responsibility. Also, the function need to provide the research needed to back up proposed measures for solving the identified problems (DSH 1989).

In practice, it means the continued development and actions for a more holistic, cross-disciplinary, transboundary coordinated and as appropriate integrated approach to the use and protection of the seas and the adjacent river basins. This, with care for the sustainability of marine ecosystems (O’Boyle and Jamieson 2006; Ottersen et al. 2011; Skern-Mauritzen et al. 2016).

Today, this need of cooperation and coordination receives serious attention in ocean governance and is highlighted in most marine international frameworks (Carneiro 2013; Valman 2014), for instance:

- Within HELCOM the *Environment/Fish Forum* has been established as a platform for communication and collaborative actions between fisheries and environmental authorities;
- A similar forum is the HELCOM Agriculture and Environment Forum (HELCOM AGRI/ENV);
- The cooperation on MSP between HELCOM and VASAB;
- The arrangement between NEAFC and OSPAR regarding the collective management of high seas protected areas in the North East Atlantic; and
- The arrangement to make UNEP Regional Seas Programmes, Regional Fishery Bodies and Large Marine Ecosystem^14^ Mechanisms to work better together (UNEP 2016).

In recent years, the strong developments in marine technology have contributed to increased public and media interest in the marine environment and underwater life, including web-based social networks. This has increased the public awareness of and concern for the marine environment (Voyer et al. 2012). Well-informed citizens are crucial for a country’s ability to properly deal with its environmental problems (Fletcher et al. 2009). Today, the involvement of a more informed public, including NGOs, in how the marine environment is managed, has increased the pressure on concerned international organizations and responsible national authorities on how our oceans and seas are managed.

## Conclusions and suggestions for improved marine governance

International and national marine environmental governance need well-functioning organizations and legal frameworks as a basis for action and in support of responsible and effective marine and maritime management by individual countries, as emphasized in the following:

- Future marine and maritime management needs even greater emphasis on international cooperation through well-functioning multilateral organisations. This, requires relevant mandates by national governments to take on board global or regional processes, expert roles and normative frameworks;
- The United Nations Convention on the Law of the Sea and Chapter 17 of Agenda 21 has become the overall legal and programme framework for ocean affaires (see “Intergovernmental and non-governmental organizations outside the UN system” section). However, to make it effective, present shortcomings of the system have to be resolved and realistic global, regional and national maritime policies with clear targets and timetables need to be developed and agreed upon. In this respect MSP, ICAM and ecosystem-based management—if properly developed, legally implemented and effectively enforced—are management instruments that can contribute to a more coherent, multi-sectorally coordinated management of the use, conservation and protection of the marine and coastal environment and its resources, including freshwater catchments;
- Beside the global, legal and institutional frameworks for ocean affairs the importance of regional organisations and conventions within and outside the UN system has grown as bases for action. In fact, implementation of several environmental global programmes is carried out at a regional level by organisations such as HELCOM, OSPAR, the Regional Seas Programmes of UNEP and the regional fisheries organizations related to FAO. The regional programmes and their Contracting Parties are closer to the problems. They can often deal more effectively with the regional specificities, capabilities and perceived priorities, for instance, regarding measures to reduce pollution and establish marine protected areas;
- Although the assessment capacity is strong in many regions, there is a clear need to develop greater expertise and infrastructure around the globe in the technical aspects of marine assessment. Not least, there is a need to develop new and more consistent ways to value environmental goods and services, and internalize such valuing requirements in sector legislation (Kill 2015); and
- The further development of the underwater marine technology facilitates opportunities to increase information and communication on the marine environment its problems and values. This will in turn increase the public awareness of marine and maritime issues, as well as the possibility of NGOs to influence and put pressure on countries’ policies and practices for marine and maritime management.

## Abbreviations

CBD: Convention on Biological Diversity
COP: Conference of the Parties
DG: Directorate-General
EC: European Commission
EU: European Union
HELCOM: Convention for the Protection of the Marine Environment of the Baltic Sea Area
ICAM: Integrated Coastal Area Management
ICES: International Council for Exploration of the Sea
MARE: Maritime Affaires and Fisheries
MSFD: Marine Strategy Framework Directive
MSP: Marine Spatial Planning
OSPAR: Convention for the Protection of the Marine Environment of the North East Atlantic
PITF: Programme Implementation Task Force
RSP: Regional Seas Programme
UNCED: United Nations Conference on the Environment and Development
UNCLOS: United Nations Convention on Law of the Sea
UNEP-CAR/RCU: United Nations Environment Programme, Caribbean/Regional Coordinating Unit
VASAB: Vision and Strategies around the Baltic Sea

## Footnotes

1 A legal framework consists of the aggregation of laws enacted over time by the legislative authorities of a country, plus the common law and customary law, which have been accumulated through judicial or traditional practice.

2 The institutional framework is the systems of institutions referring to all public and civil society organizations contributing to the implementation of a certain policy objective and responsible for managing, conserving, using public goods and services provided by the resources concerned.

3 UNCLOS I was held in 1956 in Geneva, UNCLOS II in 1960 in Geneva and UNCLOS III in New York, 1973–1982.

4 Sweden is different. The Swedish territory, out to the territorial boundary, is divided into municipalities. It means that the responsibility of a coastal municipality covers coastal waters out to the territorial boundary. Also, in Sweden a private land property can include water out to around 300 m from the shoreline (DSH 1983).

5 The term "soft law" refers to half-legal instruments which do not have any legally binding force, or whose binding force is somewhat "weaker" than the binding force of traditional law. A soft law you can agree on, but need not necessarily follow.

6 The ecosystem approach is primarily concerned with sustainable environmental management at a systems level rather than focusing on individual species or habitats. It aims to take into account both the environmental and social contexts and thus provide a more integrated management methodology (Convention on Biological Diversity COP 5).

7 Ecosystem-based management is an environmental management approach to maintaining or restoring the composition, structure, function, and delivery of services of natural and modified ecosystems for the goal of achieving sustainability. It is based on an adaptive, collaboratively developed vision of desired future conditions that integrates ecological, socio-economic, and institutional perspectives, applied within a geographic framework, and defined primarily by natural ecological boundaries. It has developed more recently for the marine environment (Millennium Ecosystem Assessment 2005).

8 The Helsinki Commission (HELCOM) is the secretariat for the Helsinki Convention.

9 The OSPAR Commission is the secretariat for the OSPAR Convention that is the merged former Oslo and Paris conventions.

10 Governance is the establishment of policies and strategies, and continuous monitoring of their proper implementation, by the members of the governing body of an organization.

11 Management in organizations is the function that coordinates the efforts of people to accomplish goals and objectives using available resources efficiently and effectively.

12 Marine refer to studies of the oceans and seas including their flora and fauna as well as their interaction with coastal territories and with the atmosphere. One of the major concerns of marine research is the preservation of marine ecosystems.

13 Maritime refer to industries/livelihoods related to the human use of ocean and seas resources, involving shipping, fishery, tourism, design, construction etc.

14 Large marine ecosystems (LMEs) are regions of the world's oceans, encompassing coastal areas from river basins and estuaries to the seaward boundaries of continental shelves and the outer margins of the major ocean current systems. They are relatively large regions on the order of 200 000 km² or greater. The system of LMEs has been developed by the US National Oceanic and Atmospheric Administration (NOAA). The objective is to use the LME concept as a tool for enabling ecosystem-based management to provide a collaborative approach to management of resources within ecologically bounded transnational areas.
